# Selection and Evaluation of Tissue Specific Reference Genes in *Lucilia sericata* during an Immune Challenge

**DOI:** 10.1371/journal.pone.0135093

**Published:** 2015-08-07

**Authors:** Andre Baumann, Rüdiger Lehmann, Annika Beckert, Andreas Vilcinskas, Zdeněk Franta

**Affiliations:** 1 Department of Bioresources, Fraunhofer Institute for Molecular Biology and Applied Ecology, Giessen, Germany; 2 Institute of Phytopathology and Applied Zoology, Justus-Liebig-University of Giessen, Giessen, Germany; University of Lleida, SPAIN

## Abstract

The larvae of the common green bottle fly *Lucilia sericata* (Diptera: *Calliphoridae*) have been used for centuries to promote wound healing, but the molecular basis of their antimicrobial, debridement and healing functions remains largely unknown. The analysis of differential gene expression in specific larval tissues before and after immune challenge could be used to identify key molecular factors, but the most sensitive and reproducible method qRT-PCR requires validated reference genes. We therefore selected 10 candidate reference genes encoding products from different functional classes (*18S rRNA*, *28S rRNA*, *actin*, *β-tubulin*, *RPS3*, *RPLP0*, *EF1α*, *PKA*, *GAPDH* and *GST1*). Two widely applied algorithms (GeNorm and Normfinder) were used to analyze reference gene candidates in different larval tissues associated with secretion, digestion, and antimicrobial activity (midgut, hindgut, salivary glands, crop and fat body). The Gram-negative bacterium *Pseudomonas aeruginosa* was then used to boost the larval immune system and the stability of reference gene expression was tested in comparison to three immune genes (*lucimycin*, *defensin-1* and *attacin-2*), which target different pathogen classes. We observed no differential expression of the antifungal peptide *lucimycin*, whereas the representative targeting Gram-positive bacteria (*defensin-1*) was upregulated in salivary glands, crop, nerve ganglion and reached its maximum in fat body (up to 300-fold). The strongest upregulation in all immune challenged tissues (over 50,000-fold induction in the fat body) was monitored for *attacin-2*, the representative targeting Gram-negative bacteria. Here we identified and validated a set of reference genes that allows the accurate normalization of gene expression in specific tissues of *L*. *sericata* after immune challenge.

## Introduction

Maggot debridement therapy (MDT) was established at the beginning of the 20^th^ century and became a popular treatment for chronic and recalcitrant wounds. The advent of antibiotics made MDT largely obsolete [1], but intractable wounds still present a challenge for modern medicine and MDT has re-emerged as an alternative therapeutic approach [2,3]. One hallmark of the renewed popularity of MDT is its approval as a medical device by the United States Food and Drug Administration in 2004 (FDA case number K033391), and sterile larvae of the common green bottle fly *Lucilia sericata* (Diptera: *Calliphoridae*) have therefore become the exclusive species used for MDT.

Sterile maggots are applied to a wound bed where they debride the necrotic tissue, disinfect the wound and promote wound healing [4,5]. Debridement is accomplished by the secretion of enzymes such as collagenases [6], proteases [7] and nucleases [8], which liquefy the tissue and allow the uptake of nutrients by the maggots. Some of these enzymes can also inhibit or degrade biofilms [8,9,10].

Disinfection is facilitated by the uptake and digestion of microorganisms [11], and by the secretion of antimicrobial metabolites [12,13,14] (e.g. Seraticin) and a variety of antimicrobial peptides (AMPs) [15,16,17,18]. These molecules have the potential to address the emergence and spread of antibiotic resistant pathogens [19]. Indeed, maggot secretions have shown promising activities against clinically relevant strains of drug-resistant pathogens and provide a source of lead structures for new antibiotics [13,14,17]. The antimicrobial activity of maggot secretions has a dose-dependent relationship according to the number and nature of bacteria the larvae encounter [20,21,22]. The secretions of sterile larvae do not possess antimicrobial activity whereas larvae confronted with *Staphylococcus aureus* or *Pseudomonas aeruginosa* (the most prominent Gram-positive and Gram-negative bacteria in chronic wounds, respectively [23,24,25,26,27,28]) produce distinct antimicrobial secretions that are not mutually antagonistic [20]. Both bacteria are well known to form biofilms, which protect them against external cues and considerably hamper the treatment [29,30,31].

MDT can also stimulate cell growth and wound healing but this is poorly understood. Maggot secretions can suppress pro-inflammatory responses [32,33], induce blood clotting [34], encourage fibroblasts to spread through the wound [35,36] and regulate the activation of human complement system [37]. Medical maggots also secrete allantoin and urea, which are thought to promote wound healing and are included in a number of medical products [38,39].

Although the molecular basis of MDT has been investigated, little is known about the key molecules responsible for these processes. Next-generation sequencing provides a rapid approach for the generation of genome and transcriptome datasets from non-model organisms such as *L*. *sericata*. These datasets allow the identification of medically and industrially relevant genes. To precisely characterize these genes based on their differing expression profiles in specific tissues or under specific conditions other approaches are necessary. The quantitative reverse transcription real-time polymerase chain reaction (qRT-PCR) is a powerful and sensitive method for the quantitation of gene expression [40,41] but is highly dependent on appropriate normalization to correct systemic variations [42,43,44]. The most reliable normalization is achieved by comparing gene expression profiles to stably-expressed reference genes [43,45,46]. A universal reference gene that remains stable under all conditions is unlikely to exist, so panels of candidate reference genes must be validated for certain experimental settings, such as stability during an immune challenge experiment. Here we investigated a panel of 10 candidate *L*. *sericata* reference genes in different tissues to allow the normalization of gene expression data in immune challenge experiments, to validate the identification of genes involved in maggot therapy.

## Materials and Methods

### Maggot rearing

*L*. *sericata* larvae were obtained from BioMonde GmbH (Barsbüttel, Germany). First instar larvae were reared on Columbia Agar with Sheep Blood PLUS (Thermo Scientific Oxoid) for 72 hours (h) at 28°C in the dark. The fed larvae were cleaned using sterile water before immune challenge or dissection.

### Immune challenge and zone of inhibition assay*s*

Larvae were placed in Petri dishes on ice to reduce motility and facilitate injection of larvae with *Pseudomonas aeruginosa*. The dorsal posterior was pricked with a sterile needle (wounded) or with a needle dipped in *P*. *aeruginosa* (DSM 50071) suspension (OD_600_ = 60) in phosphate buffered saline (immune-challenged). Treated larvae were supplied with fresh blood agar and incubated for additional 24 h at 28°C in the dark followed by the dissection of individual tissues. For the zone of inhibition assays, 7 ml of 1% LB agar per plate was cooled to 42°C, supplemented with 7 μl of fresh *E*. *coli* D31 [47] culture (OD_600_ = 0.5) and poured into a Petri dish, before 3-mm wells were stamped into the agar using a sterile hole puncher. After 24 h equal volumes (3 μl) of hemolymph from naïve, wounded and immune-challenged larvae were collected and immediately applied to the agar wells. The plates were incubated at 37°C for 24 h and 3 μl of 100 mg/ml ampicillin were used as a positive control.

### Tissue dissection, RNA isolation and cDNA synthesis

Larvae were cooled on ice and dissected by ripping the dorso-anterior cuticle in sterile DEPC-treated PBS under a binocular microscope. The midgut, hindgut, salivary glands, crop, fat body and nerve ganglion were harvested following Freeman and Bracegirdle [48] and equivalent amounts of tissue-specific RNA were collected by preparing three pools of midgut (n = 5), hindgut (n = 10), salivary glands, crop, fat body and nerve ganglion (each n = 25) in RA1 buffer (Macherey-Nagel) before RNA isolation.

Total RNA from naïve and immune-challenged larvae (n = 5) subsequently pooled in equimolar RNA amounts, as well as the dissected larval tissues listed above, were extracted using the NucleoSpin RNA kit (Macherey-Nagel) including a 15-min DNase I on-column digest. The concentration, purity and quality of the RNA were determined by spectrophotometry (Take3, BioTek) and agarose gel electrophoresis (S1 Fig). Only samples with A_260_/A_280_ and A_260_/A_230_ > 1.8 and at least one sharp band representing *18S rRNA* were used for cDNA synthesis. RNA samples that did not meet these criteria were cleaned and concentrated by sodium acetate precipitation [49].

Complementary DNA was synthesized using 1.5 μg of total RNA, oligo(dT)_18_ primers and the First Strand cDNA Synthesis Kit (Thermo Fisher Scientific) according to manufacturer’s recommendations. The resulting cDNA was diluted to a working concentration of 400 pg/μl, divided into aliquots and stored at –80°C.

### Candidate gene sequence assembly

*L*. *sericata* reference gene candidates were selected based on known reference genes from the closely-related species *Lucilia cuprina* [50] and other arthropods [51,52]. The peptide sequences of each gene (or the nucleotide sequences of *18S* and *28S* rRNA) were queried against the *L*. *sericata* transcriptome database [15] using the BLAST algorithm. BLASTn was used to find all reads matching the search results and the pool of these reads was expanded to include further paired-end read partners. The final group of reads was assembled using *Trinity* [53] and this step was repeated until the complete coding sequences were acquired. Finally, *Gap5* [54] was used to verify the finished sequences with mapped reads assembled by *Bowtie* [55].

### Primer design and evaluation

Gene-specific primers were designed using Oligo Explorer v1.1.2 (http://www.softpedia.com/get/Science-CAD/Oligo-Explorer.shtml) to yield primers 19–23 nucleotides in length with amplification products of 50–210 bp and T_m_ values of ~60°C. All primers were subsequently tested in a standard curve assay including melt curve analysis using the StepOnePlus Real-Time PCR System (Applied Biosystems). The cDNA concentration ranging from 50 ng to 3.2 pg of total RNA was used in 5-fold dilutions with the cycling conditions recommended by the manufacturer. Briefly, hot-start PCR with denaturation at 95°C for 10 min was followed by 40 cycles of 95°C for 15 s and 60°C for 60 s, and finally the melt curve analysis with a temperature increase from 60°C to 95°C in 0.5°C steps. Reaction efficiency was calculated using StepOne Software v2.3 and only primers with an efficiency of 90–110%, R^2^ ≥ 0.99 and a single sharp melt curve peak were used for reference gene evaluation.

### Quantitative real-time RT-PCR

Quantitative real-time RT-PCR was carried out on a StepOnePlus system (Applied Biosystems) using optical 96-well plates (Applied Biosystems). The total reaction volume of 10 μl contained 5 μl Power SYBR Green PCR Master Mix (Applied Biosystems), 1 μl (400 pg) cDNA and 300 nM of each primer except for *β-tubulin* (150 nM), *EF1α* (150 nM) and *actin* (450 nM forward primer and 150 nM reverse primer). All reactions were carried out in three technical replicates under the reaction conditions stated above. Baseline correction was performed automatically by StepOne Software v2.3 and the quantification cycle (Cq) was always determined at an intensity of 0.15. All cDNA samples and corresponding RNA without reverse transcription (no-RT control) were tested with the *18S rRNA* primers to estimate the remnants of genomic DNA [56] and only samples with a ΔCq ≥ 10 were accepted.

### Normfinder and GeNorm analysis

The Normfinder and GeNorm algorithms were used for reference gene assessment. Normfinder [57] was used as an add-in for Microsoft Excel (http://moma.dk/normfinder-software), and GeNorm [46] was used as part of the R package NormqPCR with R version 3.1.1 [58]. In both cases, raw Cq values were used and log transfer was performed by the software.

### Analysis of gene expression following immune challenge

Gene-specific primers were designed for the coding sequence of three *L*. *sericata* immune genes *lucimycin*, *defensin-1* and *attacin-2*, which are differentially expressed when the larval immune system is challenged [15]. Genes were chosen based on their activity spectrum, with lucimycin targeting fungi [16], defensin-1 targeting Gram-positive [59] and attacin-2 targeting Gram-negative bacteria [59], respectively. Gene expression profiles upon immune challenge were determined by qRT-PCR using the ΔΔCq method [60] in Rest2009 (http://www.gene-quantification.de/rest-2009.html). The data were normalized using three different combinations of genes from the reference gene assessment: (a) the two best reference genes for every sample (GeNorm); (b) the three overall best reference genes (GeNorm and Normfinder); and (c) the six overall best reference genes (GeNorm). Efficiencies greater than 100% were set to 100% because Rest2009 does not allow higher values.

## Results

### Selection of candidate reference genes

We monitored the expression level of 10 *L*. *sericata* candidate reference genes before and after challenging the larvae with *P*. *aeruginosa* (Table 1). Our goal was to identify reference genes appropriate for different larval tissues, with the key criterion that gene expression is not affected by immune challenge. The tissues were selected based on their role in secretion and/or digestion (salivary glands, crop, midgut and hindgut) and supplemented by additional tissues (nerve ganglion and fat body). Candidates were chosen based on earlier studies in arthropods using orthologs of these genes [50,52]. To minimize the risk of co-regulation, we selected genes representing different functional classes of proteins. The *L*. *sericata* transcriptome database was explored to identify orthologs and the coding sequences of the 10 selected candidate reference genes were submitted to GenBank (Table 1).

**Table 1:** Genes and qRT-PCR primers evaluated in this study.

Gene name	Abbreviation	Accession no.	Primer sequences (5'-3')	L (bp)^a^	E (%)^b^	R^2^ ^c^
*18S ribosomal RNA*	*18S rRNA*	KR133393*	Fwd 5' AGCAGTTTGGGGGCATTAG 3'	171	94	0.996
			Rev 5' GCTGGCATCGTTTATGGTTAG 3'			
*28S ribosomal RNA*	*28S rRNA*	KR133394*	Fwd 5' CCAAAGAGTCGTGTTGCTTG 3'	180	91	0.997
			Rev 5' ATTCAGGTTCATCGGGCTTA 3'			
*40S ribosomal protein S3*	*RPS3*	KR133395*	Fwd 5' TCAAGGTGTTTTGGGTATCAAGG 3'	156	90	0.999
			Rev 5' GCGGGCATTTTGTATTCTGTTTC 3'			
*Elongation factor 1-alpha 1*	*EF1α*	KR133396*	Fwd 5' TGTCGGTGTCAACAAGATGG 3'	137	93	0.998
			Rev 5' GAGATGGGAACGAAGGCAAC 3'			
*60S acidic ribosomal protein P0*	*RPLP0*	KR133397*	Fwd 5' GGTGCTGATAATGTTGGTTC 3'	78	101	0.995
			Rev 5' ACCCATAAGGACGACACC 3'			
*actin*	*actin*	KR133398*	Fwd 5' TGCCGATCGTATGCAAAA 3'	90	100	0.998
			Rev 5' ACGGAGTATTTGCGTTCTGG 3'			
*Beta-1-tubulin*	*ß-tubulin*	KR133399*	Fwd 5' AAACTAACCACACCCACATACGG 3'	173	90	0.999
			Rev 5' AGAGGAGCAAAACCAGGCAT 3'			
*cAMP-dependent protein kinase*	*PKA*	KR133400*	Fwd 5' CAACACAAGCCGACAAAAGAC 3'	145	106	0.995
			Rev 5' GATAGCGTAGGGAAACCAAGAA 3'			
*Glyceraldehyde-3-phosphate dehydrogenase 1*	*GAPDH*	KR133401*	Fwd 5' GAACGGCAAACTCACTGGTATG 3'	182	104	0.997
			Rev 5' CGGTGGAAACGACTTCTTCATC 3'			
*Glutathione S-transferases 1–1*	*GST1*	KR133402*	Fwd 5' GCCAGTGTCAGCACCTTCG 3'	120	92	0.999
			Rev 5' GCAACCTTCCCAGTTTTCATC 3'			
*attacin-2*	*atta2*	KR920003*	Fwd 5' GCACCTTAGCCTACAATAACAATGG 3'	92	103	0.999
			Rev 5' ACTGATGCTCTTGGTCAAAGTATCG 3'			
*defensin-1*	*def1*	KT149727*	Fwd 5' CGGAGTTACATGGTCGTTACAAGAG 3'	164	109	0.993
			Rev 5' CGGTGTCCAATCAACAAACAGTG 3'			
*lucimycin*	*afp*	KJ413251	Fwd 5' TCGCTTTAATCGCCGTTGTT 3'	103	96	0.999
			Rev 5' ATGATGCCCAGCCTGTTGTTC 3'			

*Indicates GenBank submission of sequence obtained in the present study.

^a^Length of amplicon.

^b^Quantitative RT-PCR efficiency.

^c^Coefficient of determination.

### Quantitative RT-PCR

**Fig 1:**
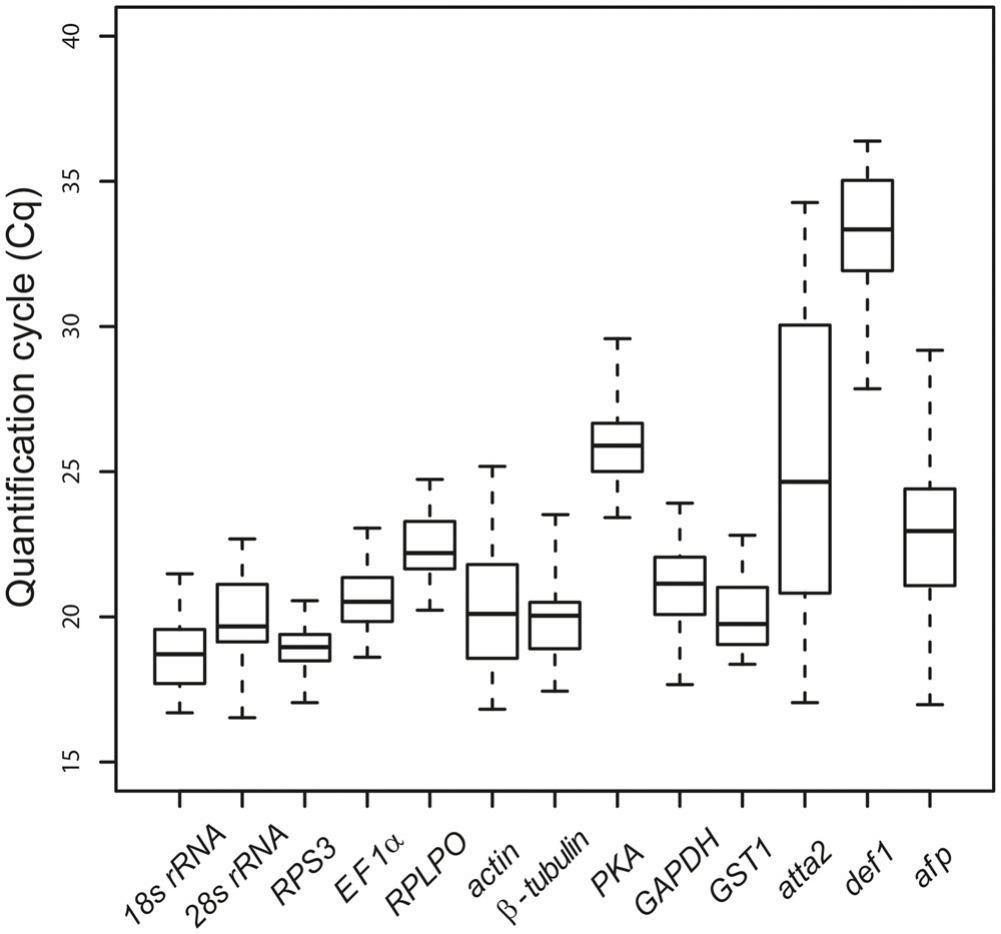
Distribution of quantification cycle (Cq) values for all *L*. *sericata* candidate genes obtained by qRT-PCR. Data for all naïve and immune-challenged samples were pooled (n = 42). Boxplots show first to third quartile of values in the box, the center line indicates the median, vertical dotted bars extend to the highest and lowest value.

The efficiencies of each qRT-PCR primer pair were generally high. Based on the standard curve slopes determined using StepOne software, the efficiencies ranged from 90% to 109% with R^2^ values > 0.99 for all pairs (Table 1). Linear behavior was observed over a concentration range spanning four orders of magnitude (50 ng to 3.2 pg of cDNA). The absence of primer dimers was confirmed by a melting curve analysis, which resulted in only one sharp peak for each amplicon under our experimental conditions (S2 Fig). All 10 reference genes were expressed in all naïve and immune-challenged samples. Median expression values ranged from 19 Cq (*18S rRNA*) to 26 Cq (*PKA*) and standard deviations ranged from 0.75 Cq (*RPS3*) to 2.1 Cq (*actin*) as shown in Fig 1.

### Zone of inhibition assays and initial analysis of *attacin-2* expression

To monitor the changes in larval reference gene expression triggered by interaction with bacteria, we chose the opportunistic pathogen *P*. *aeruginosa* and direct infection by pricking the *L*. *sericata* larvae with a bacteria-coated needle [61]. *P*. *aeruginosa* has previously been used to induce an immune response [15] and is also a common pathogen found in chronic wounds. Two independent tests were used to monitor the success of the immune challenge.

**Fig 2:**
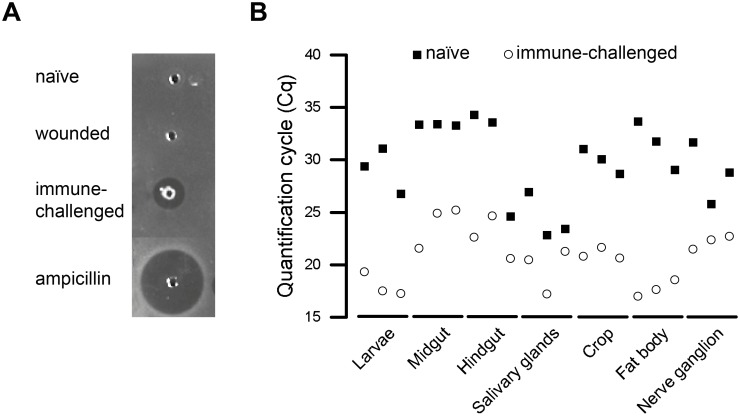
Validation of *Pseudomonas aeruginosa* immune challenge. **A:** Samples of hemolymph from naïve, wounded and immune-challenged larvae were tested in the *E*. *coli* zone of inhibition assay, with 100 mg/ml ampicillin as a positive control. Only immune-challenged larvae generated a zone of inhibition. **B:** Distribution of quantification cycle (Cq) values for immune gene *attacin-2* in all naïve and immune-challenged samples. Raw values for all three biological replicates of the six tissue and the larvae samples are displayed separately.

The first was a zone of inhibition assay with *E*. *coli* strain D31 [47]. Hemolymph from naïve, wounded and immune-challenged larvae were applied to the agar wells and the plates were incubated at 37°C for 24 h. Only the hemolymph from immune-challenged larvae produced distinct inhibition zones (Fig 2a).

As a second test, we carried out an initial analysis of gene expression profiles to monitor changes at the RNA level triggered by immune challenge, using *attacin-2* mRNA as a marker because this AMP is known to be upregulated under similar conditions [15]. The raw Cq values for *attacin-2* mRNA from two distinct populations, i.e. the naïve samples (Cq ~30) and the immune-challenged (Cq ~20), are shown in (Fig 2b). No naïve sample showed a lower value than the corresponding immune-challenged sample. These raw data supported our initial hypothesis that *L*. *sericata attacin-2* mRNA is induced by immune challenge with a Gram-negative bacterium, but can only be validated by normalizing against the expression levels of reliable reference gene(s).

### Validation of candidate reference genes using Normfinder

The model-based Normfinder approach provides a direct estimation of variance in so-called stability values, with lower numbers equating a more stable expression. The program does not sequentially eliminate unsuitable genes so the pre-exclusion of genes with large variances is necessary to achieve reliable results.

Normfinder analysis of the 10 candidate reference genes (Table 2) ranked *RPS3*, *RPLP0* and *EF1α* as the three most stable genes considering immune challenge. The worst stability values (approximately 2-fold higher across all samples) were observed for *18S rRNA*, *actin* and *28S rRNA*. The overall ranking was not transferable to all specific tissues or to whole larvae because the stability of the candidates differed according to the sample type. Intragroup variation was generally low with only *28S rRNA* showing variation (S1 Table). Intergroup variation was generally much higher, with the highest scores generated by *18S rRNA*, *28S rRNA* and *actin* (S2 Table).

**Table 2:** Normfinder ranking of the stability values of candidate reference genes.

Rank	Larvae	Midgut	Hindgut	Salivary glands	Crop	Fat body	Nerve ganglion	Overall
1	*RPLP0*	*EF1α*	*RPLP0*	*PKA*	*β-tubulin*	*RPLP0*	*PKA*	*RPLP0*
	(0.010)	(0.010)	(0.006)	(0.005)	(0.007)	(0.006)	(0.010)	(0.027)
2	*actin*	*actin*	*EF1α*	*RPS3*	*actin*	*18S rRNA*	*GST1*	*RPS3*
	(0.017)	(0.010)	(0.012)	(0.005)	(0.011)	(0.007)	(0.011)	(0.029)
3	*GAPDH*	*GST1*	*RPS3*	*GAPDH*	*PKA*	*β-tubulin*	*actin*	*EF1α*
	(0.020)	(0.012)	(0.015)	(0.014)	(0.016)	(0.009)	(0.015)	(0.029)
4	*RPS3*	*RPLP0*	*GAPDH*	*GST1*	*RPS3*	*RPS3*	*18S rRNA*	*PKA*
	(0.021)	(0.014)	(0.018)	(0.016)	(0.019)	(0.013)	(0.017)	(0.037)
5	*GST1*	*PKA*	*actin*	*actin*	*GAPDH*	*PKA*	*RPLP0*	*GST1*
	(0.022)	(0.019)	(0.020)	(0.024)	(0.026)	(0.019)	(0.022)	(0.039)
6	*EF1α*	*18S rRNA*	*PKA*	*β-tubulin*	*GST1*	*EF1α*	*GAPDH*	*GAPDH*
	(0.025)	(0.020)	(0.022)	(0.027)	(0.027)	(0.036)	(0.029)	(0.041)
7	*PKA*	*β-tubulin*	*β-tubulin*	*EF1α*	*18S rRNA*	*GST1*	*β-tubulin*	*β-tubulin*
	(0.026)	(0.020)	(0.028)	(0.039)	(0.030)	(0.042)	(0.033)	(0.052)
8	*18S rRNA*	*GAPDH*	*18S rRNA*	*RPLP0*	*EF1α*	*GAPDH*	*RPS3*	*18S rRNA*
	(0.036)	(0.021)	(0.033)	(0.041)	(0.030)	(0.044)	(0.034)	(0.061)
9	*β-tubulin*	*RPS3*	*GST1*	*18S rRNA*	*RPLP0*	*actin*	*EF1α*	*actin*
	(0.037)	(0.031)	(0.034)	(0.057)	(0.030)	(0.056)	(0.037)	(0.068)
10	*28S rRNA*	*28S rRNA*	*28S rRNA*	*28S rRNA*	*28S rRNA*	*28S rRNA*	*28S rRNA*	*28S rRNA*
	(0.051)	(0.074)	(0.056)	(0.073)	(0.036)	(0.131)	(0.078)	(0.072)

### Validation of candidate reference genes using GeNorm

The GeNorm algorithm calculates the pairwise variation among all tested genes and assigns stability measures (M). In every cycle, the gene with the highest stability measure (i.e. the least stable) is excluded until only the two best genes remain. GeNorm analysis of our 10 candidate reference genes (Table 3) yielded similar results to Normfinder. GeNorm ranked *RPS3*, *RPLP0* and *EF1α* as the three best genes and *18S rRNA*, *actin* and *28S rRNA* as the three worst, with M values of 2 to 4-fold higher than the best candidates across all samples. As for the Normfinder data, the overall GeNorm ranking list was not transferable to all individual tissues or whole larvae.

**Table 3:** GeNorm ranking of stability measures for candidate reference genes and *attacin-2*.

Rank	Larvae	Midgut	Hindgut	Salivary glands	Crop	Fat body	Nerve ganglion	Overall
1	*RPLP0*	*β-tubulin*	*actin*	*PKA*	*β-tubulin*	*RPLP0*	*EF1α*	*EF1α*
	(0.341)	(0.270)	(0.128)	(0.209)	(0.252)	(0.357)	(0.241)	(0.557)
1	*actin*	*EF1α*	*RPS3*	*RPS3*	*RPS3*	*RPS3*	*RPLP0*	*RPLP0*
	(0.341)	(0.270)	(0.128)	(0.209)	(0.252)	(0.357)	(0.241)	(0.557)
3	*EF1α*	*PKA*	*PKA*	*GAPDH*	*actin*	*β-tubulin*	*GAPDH*	*RPS3*
	(0.403)	(0.313)	(0.366)	(0.514)	(0.308)	(0.489)	(0.418)	(0.841)
4	*GAPDH*	*RPS3*	*RPLP0*	*β-tubulin*	*GST1*	*18S rRNA*	*actin*	*GST1*
	(0.512)	(0.356)	(0.424)	(0.522)	(0.384)	(0.636)	(0.452)	(1.164)
5	*GST1*	*GAPDH*	*EF1α*	*actin*	*GAPDH*	*PKA*	*PKA*	*GAPDH*
	(0.586)	(0.427)	(0.408)	(0.536)	(0.410)	(0.778)	(0.672)	(1.154)
6	*18S rRNA*	*actin*	*GST1*	*GST1*	*PKA*	*GAPDH*	*GST1*	*PKA*
	(0.588)	(0.557)	(0.522)	(0.620)	(0.540)	(0.947)	(0.646)	(1.219)
7	*RPS3*	*RPLP0*	*β-tubulin*	*EF1α*	*18S rRNA*	*EF1α*	*18S rRNA*	*β-tubulin*
	(0.712)	(0.550)	(0.608)	(0.685)	(0.729)	(0.983)	(0.656)	(1.266)
8	*PKA*	*GST1*	*GAPDH*	*RPLP0*	*EF1α*	*GST1*	*β-tubulin*	*18S rRNA*
	(0.694)	(0.547)	(0.691)	(0.730)	(0.825)	(1.015)	(0.698)	(1.637)
9	*β-tubulin*	*18S rRNA*	*18S rRNA*	*18S rRNA*	*RPLP0*	*actin*	*RPS3*	*actin*
	(0.817)	(0.660)	(0.838)	(1.339)	(0.859)	(1.207)	(0.820)	(1.836)
10	*28S rRNA*	*28S rRNA*	*28S rRNA*	*28S rRNA*	*28S rRNA*	*28S rRNA*	*28S rRNA*	*28S rRNA*
	(1.092)	(1.490)	(1.229)	(1.528)	(0.888)	(2.645)	(1.658)	(2.095)
*atta2*	*atta2*	*atta2*	*atta2*	*atta2*	*atta2*	*atta2*	*atta2*	*atta2*
	(6.201)	(5.662)	(5.874)	(3.501)	(5.550)	(8.538)	(4.569)	(5.888)

To illustrate the differences in stability measures we also included *attacin-2*, which is strongly induced by immune-challenge and therefore expected to be unstable. As anticipated, the M value for *attacin-2* was ~20-fold higher than the most stable candidate genes in each sample (Table 3).

**Fig 3:**
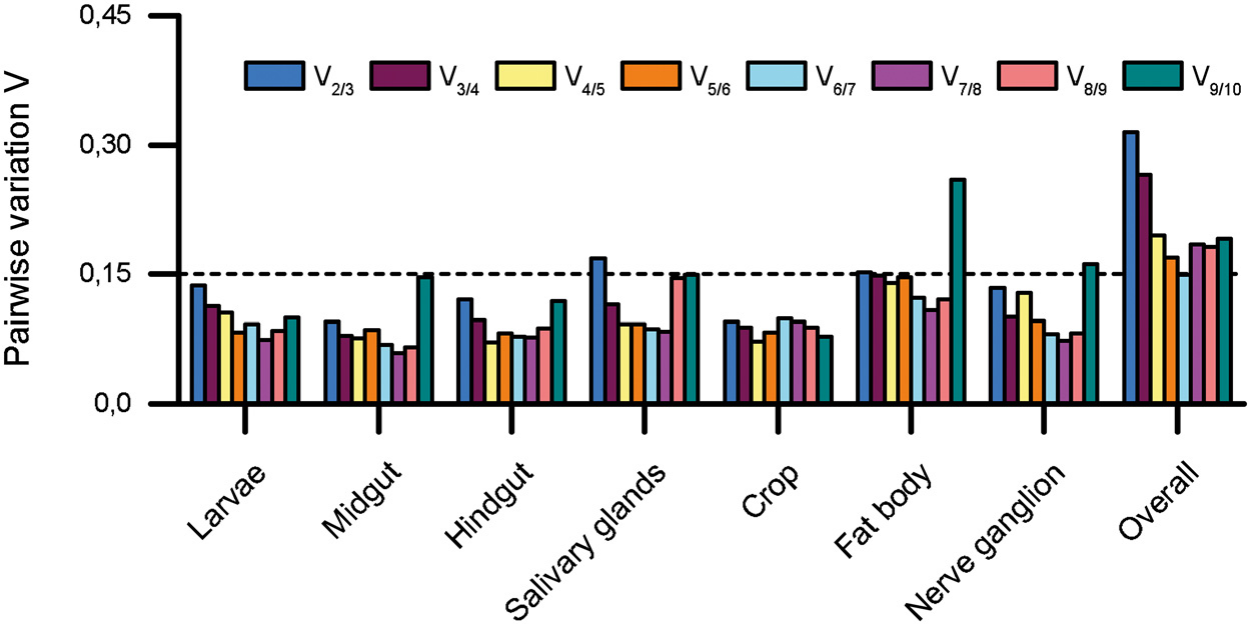
Determination of the optimal number of control genes for normalization. Pairwise variation (V_n/n+1_) analysis between the normalization factors NF_n_ and NF_n+1_ to determine the number of reference genes required for accurate normalization in every individual sample group. The dashed line at 0.15 represents the set threshold below which the number of reference genes is optimal.

Because neither algorithm selected a single reference gene suitable for all samples, we used GeNorm pairwise variation comparison and select the most appropriate number of reference genes for normalization, which is an appropriate strategy when single reference genes are unsuitable [46]. This comparison (V_n/n+1_) examines the normalization factors NF_n_ and NF_n+1_ in each analysis cycle. If the calculated value falls below a set threshold of 0.15 [46], the addition of further reference genes does not improve the quality of normalization. As shown in Fig 3, we found that most tissues reached the recommended threshold of 0.15 with just two reference genes, whereas three reference genes were required for the normalization of expression levels in the salivary glands (V_2/3_ = 0.17). When comparing overall variation, the 0.15 threshold is reached at V_6/7_ which means that six reference genes would be necessary for appropriate simultaneous normalization within all tested samples.

**Fig 4:**
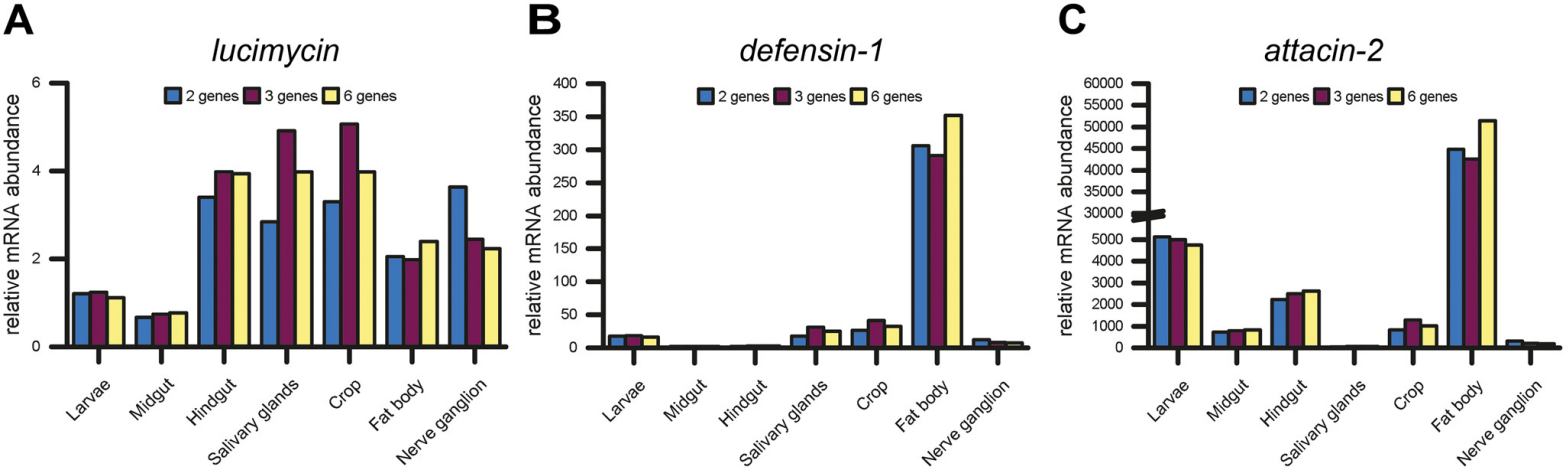
Quantitative RT-PCR analysis of *lucimycin*, *defensin-1* and *attacin-2* upon immune challenge. The mRNA expression of *lucimycin* (A), *defensin-1* (B) and *attacin-2* (C) was determined in different *L*. *sericata* tissues. Relative mRNA expression levels of individual immune genes were compared in between immune-challenged and naïve samples and normalized with two, three or six reference genes.

The Cq values for *lucimycin*, *defensin-1* and *attacin-2* mRNA were therefore normalized using three combinations of candidate reference genes: (a) the two best candidate reference genes for every sample defined by GeNorm analysis; (b) the overall best three reference genes defined by both algorithms (*RPS3*, *RPLP0* and *EF1α*); and (c) the overall best six reference genes defined by GeNorm pairwise variation comparison. Our results show that the antifungal peptide *lucimycin* is not differentially expressed (Fig 4a), whereas *defensin-1*, which is specifically targeting Gram-positive bacteria, shows different upregulation in salivary glands, crop, nerve ganglion and fat body, ranging from a 8-fold induction in the nerve ganglion to a 290-fold induction in the fat body (Fig 4b). The strongest upregulation was monitored for *attacin-2* that targets primarily Gram-negative bacteria. *Attacin-2* was strongly induced by immune challenge in all tested tissues, ranging from a 37-fold induction in the salivary glands to a 51,463-fold induction in the fat body (Fig 4c). As shown in Fig 4, all three reference gene combinations lead to similar expression results with slight variations in relative values.

## Discussion

MDT has undergone a renaissance since the end of the 20^th^ century, but although recent studies have addressed the underlying mechanisms many of the key proteins responsible for the beneficial effects of medical maggots remain unknown [4,5]. The falling cost of next generation sequencing means that the sequencing of genome and transcriptome datasets from non-model organisms can now be used as a first step to screen for medically and industrially relevant sequences. In a second step qRT-PCR can validate these candidates, but only if appropriate reference genes are available for the normalization of expression data [43,45,46]. This approach is ideal for the identification of *L*. *sericata* genes with important roles in wound healing. Thus such genes are likely to show different expression profiles in specific tissues or are modulated in the presence of wound pathogens.

We investigated the tissue specific expression stability of 10 candidate reference genes and 3 immune genes (*lucimycin*, *defensin-1* and *attacin-2*), which target different pathogen classes. We monitored the expression of these immune genes in different *L*. *sericata* tissues before and after the immune challenge with the Gram-negative bacterium *P*. *aeruginosa*, which was introduced into third-instar *L*. *sericata* larvae by pricking with a contaminated needle. We chose this invasive strategy to ensure that the comparator underwent a strong change in expression allowing the stability of the candidate reference genes to be tested robustly, even though this far exceeds the intensity of the bacterial challenge that maggots would encounter in human wounds. As expected, we monitor the strong expression of *attacin-2* (targeting Gram-negative bacteria [59]) in all larval tissues reaching its maximum in fat body (over 50,000-fold). We also did not observe any differential expression of the antifungal peptide *lucimycin*, which is known to have no effect against Gram-negative bacteria [16]. Interestingly, *defensin-1* as a representative targeting Gram-positive bacteria [59], was differentially upregulated in salivary glands, crop, nerve ganglion and fat body. This unexpected result can be easily explained by the wounding procedure itself, which has been shown to induce insect immune response previously [62]. Our results demonstrate that even under such harsh conditions, which heavily perturbed the system and led to the strong induction of *attacin-2*, the expression of most of the candidate reference genes remained stable. This success rate reflects our choice of genes that have been used as references in other insects, allowing the knowledge-based pre-selection of candidates that are likely to be suitable as reference genes in *L*. *sericata* [50,63,64,65,66,67].

The Normfinder and GeNorm algorithms were unable to identify a single optimal reference gene or an ideal combination of two to three reference genes that worked consistently across tested samples. *RPLP0*, *EF1α* and *RPS3* were identified as the most stable reference genes across all samples even though each of them ranked low for stability in at least one sample. They are all involved in protein expression [68,69,70] and co-regulation cannot be ruled out as explanation for their high ranking. However, comparison with the other candidate genes does not indicate generally co-regulated expression. Although it may be necessary to use specific candidates for particular tissues to achieve the most accurate data normalization, the use of these overall three best reference genes should be more than adequate for most experimental scenarios, as illustrated by our expression analysis of three immune genes (Fig 4).

Among the remaining candidates, some (e.g. *GST1* and *GAPDH*) occupy mid-ranking positions in many tissues and have poor scores in others but never rank as the most suitable reference gene, whereas others (e.g. *PKA* and *β-tubulin*) also occupy mid-raking position in many tissues but are the most stable reference genes in others, i.e. the salivary glands and the nerve ganglion (*PKA*) or the crop (*β-tubulin*). Their overall mid-to-low ranking reflects their tendency to show good scores in a few tissues but poor scores in most others.

Remarkably, *actin* has a low overall score but good scores in several tissues (second most stable reference in three tissues and third in another). However, the standard deviation of 2.1 Cq is the highest among all the tested candidate genes, reflecting high intergroup variation (S1 Table) rather than changes in expression induced by the immune challenge, which would lead to higher intragroup variations. It is not surprising that a fundamental structural protein like actin would be expressed at different levels in diverse tissues such as the gut and the fat body. Therefore, despite its low overall ranking, *actin* would be useful as a reference gene in most individual tissues but not in more diverse collections of samples.

Compared to the other candidates, the genes for *18S* and *28S rRNA* were the least stable and therefore the least suitable for normalization, which contrasts with the findings in other arthropods [50,71]. This may be a species-dependent phenomenon, but may also reflect the technical approach, e.g. the method used for RNA isolation and cDNA synthesis. For example, we used oligo(dT)_18_ primers which only partly reverse transcribe the rRNA genes due to the lack of a canonical polyadenylate tail.

While dissecting the fat body from immune-challenged larvae we observed the remodeling and/or degradation of this tissue. The physical appearance of the fat body changed from clusters of round cells to loosely-associated amorphous cells which yielded lower amounts of total RNA with poorer quality compared to the other tissues. After immune challenge, fat body total RNA from all biological replicates had to be precipitated and concentrated to meet the required criteria for cDNA synthesis. The insect fat body is responsible for AMP production [72] and two defense pathways have been described in *Drosophila melanogaster*, i.e. the Toll pathway against Gram-positive species and the immune deficiency (IMD) pathway against Gram-negative species [73]. Constitutive triggering of the IMD pathway has been shown to induce apoptosis [74]. Our results show that although *attacin-2* is expressed ubiquitously and globally upregulated by immune challenge, the strongest induction (over 50,000-fold) occurs in the fat body. We used *P*. *aeruginosa*, which as a Gram-negative species is targeted by attacins [59] and we hypothesize that the IMD pathway plays a major role in this immune response. The induction of *attacin-2* may therefore be part of this IMD pathway response, ultimately resulting in apoptosis which may explain the physical changes in the fat body we observed.

There is probably no universally ideal reference gene in any experimental system so it is important to distinguish between the best gene and combination of genes suitable for different experimental settings. Despite the diverse expression profiles of the 10 candidate reference genes in our dissected tissue samples, several combinations of these genes gave identical results when used for the normalization of immune genes expression during an immune challenge, suggesting that the analysis of six reference genes per sample or different reference genes in each tissue is unnecessary. From our own experience, the reliable normalization of *L*. *sericata* expression data among all dissected tissues (Fig 4) is also achieved using the three best reference genes based on our overall rankings (*RPLP0*, *EF1α* and *RPS3)*, which balances accuracy with convenience and the cost of additional experiments. The primer pairs and reference gene candidates evaluated in this study provided a valuable tool for the normalization of gene expression data in medical maggots, which will facilitate the identification and functional analysis of genes which are responsible for the beneficial effects of maggot therapy.

## Supporting Information

S1 Fig Agarose gel of purified RNA samples. RNA from all *L*. *sericata* tissues from naïve (top) and immune-challenged (bottom) larvae was analyzed using agarose gel with exception of one “crop” sample and three “fat body” samples in immune-challenged larvae. These samples were sodium acetate precipitated after the RNA isolation and limited in sample amount.
(TIF)

S2 Fig Melt curves of all applied primer pairs. (PDF)

S1 Table Normfinder intergroup variation for all candidate genes. (DOCX)

S2 Table Normfinder intragroup variation for all canditate genes. (DOCX)
