# Supplementary figures and images for: Selection and Evaluation of Tissue Specific Reference Genes in Lucilia sericata during an Immune Challenge
Source: PLoS One. 2015 Aug 7;10(8):e0135093. doi: 10.1371/journal.pone.0135093 (PMC4529112; doi:10.1371/journal.pone.0135093)

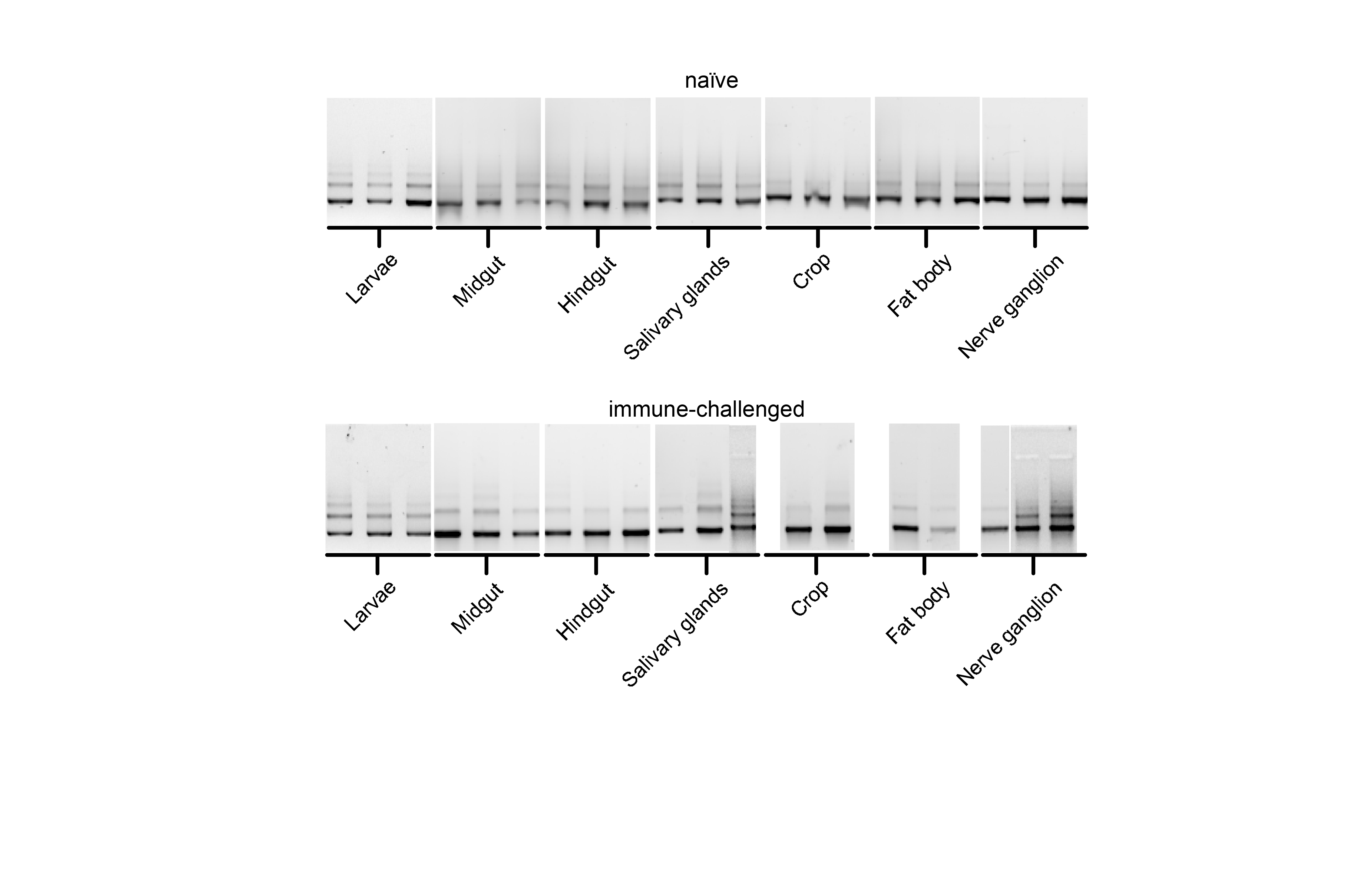

Supplement: S1 Fig — RNA from all L. sericata tissues from naïve (top) and immune-challenged (bottom) larvae was analyzed using agarose gel with exception of one “crop” sample and three “fat body” samples in immune-challenged larvae. These samples were sodium acetate precipitated after the RNA isolation and limited in sample amount. (TIF) [file pone.0135093.s001.tif]
