# Supplementary material for: Selection and Evaluation of Tissue Specific Reference Genes in Lucilia sericata during an Immune Challenge
Source: PLoS One. 2015 Aug 7;10(8):e0135093. doi: 10.1371/journal.pone.0135093 (PMC4529112; doi:10.1371/journal.pone.0135093)
Supplement: S2 Fig — (PDF) [file pone.0135093.s002.pdf]

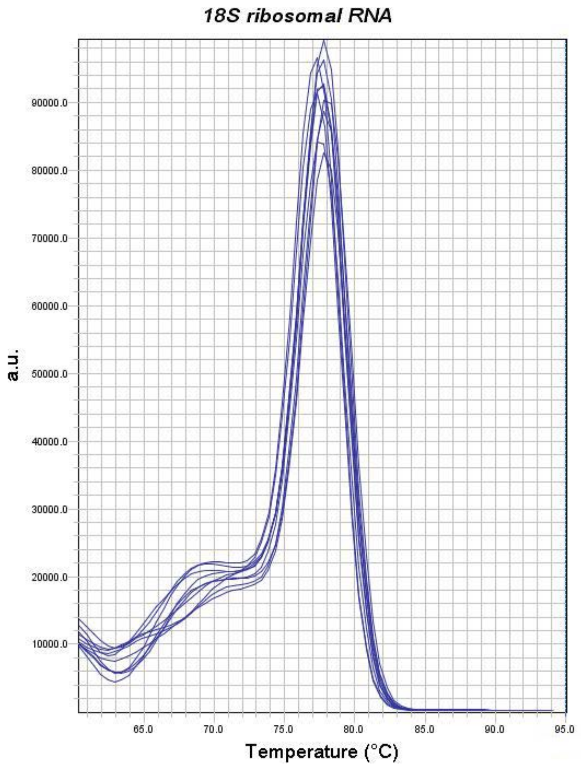
18S ribosomal RNA
a.u.
90000.0
80000.0
70000.0
60000.0
50000.0
40000.0
30000.0
20000.0
10000.0
65.0
70.0
75.0
80.0
85.0
90.0
95.0
Temperature (°C)

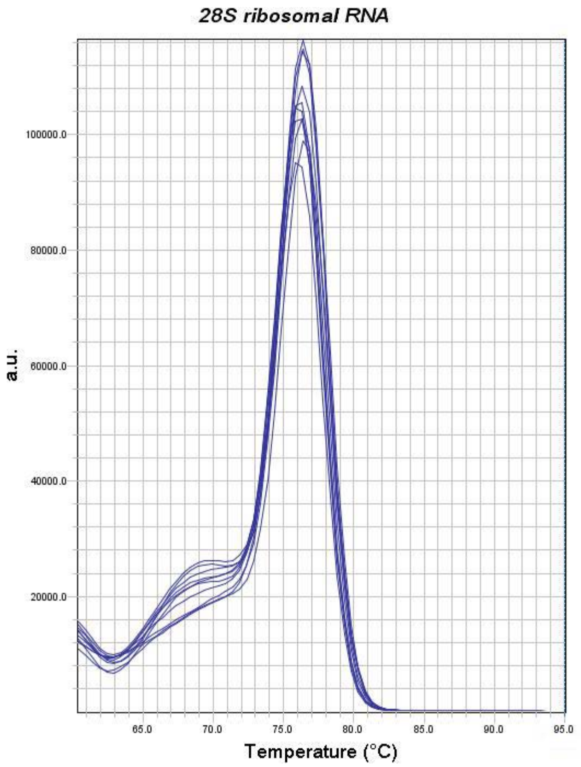
28S ribosomal RNA
a.u.
100000.0
80000.0
60000.0
40000.0
20000.0
65.0
70.0
75.0
80.0
85.0
90.0
95.0
Temperature (°C)

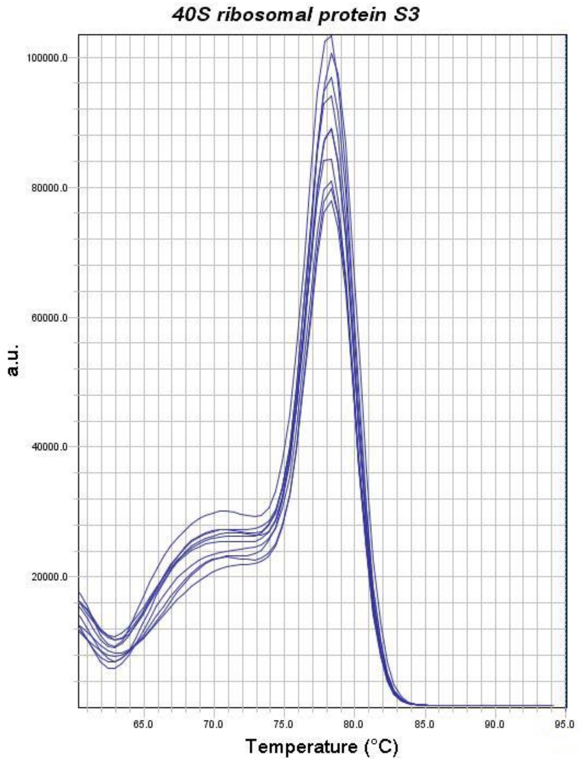
40S ribosomal protein S3
a.u.
100000.0
80000.0
60000.0
40000.0
20000.0
65.0
70.0
75.0
80.0
85.0
90.0
95.0
Temperature (°C)

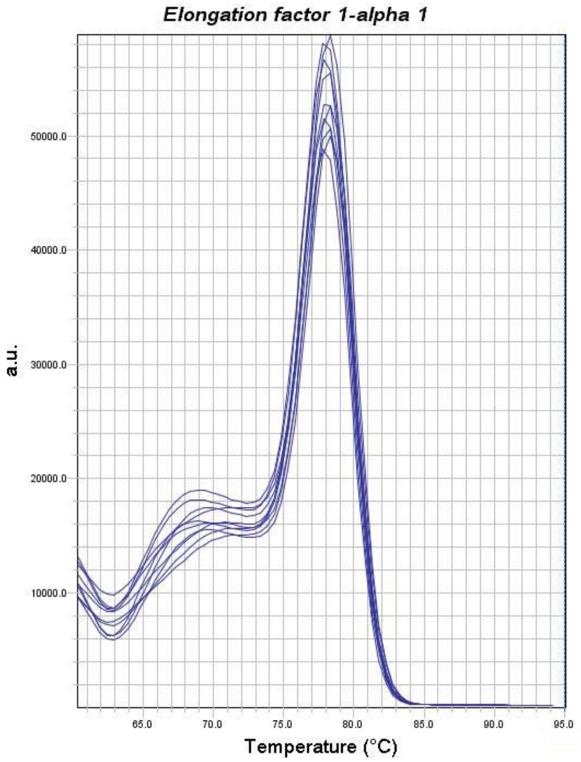
Elongation factor 1-alpha 1
a.u.
50000.0
40000.0
30000.0
20000.0
10000.0
65.0
70.0
75.0
80.0
85.0
90.0
95.0
Temperature (°C)

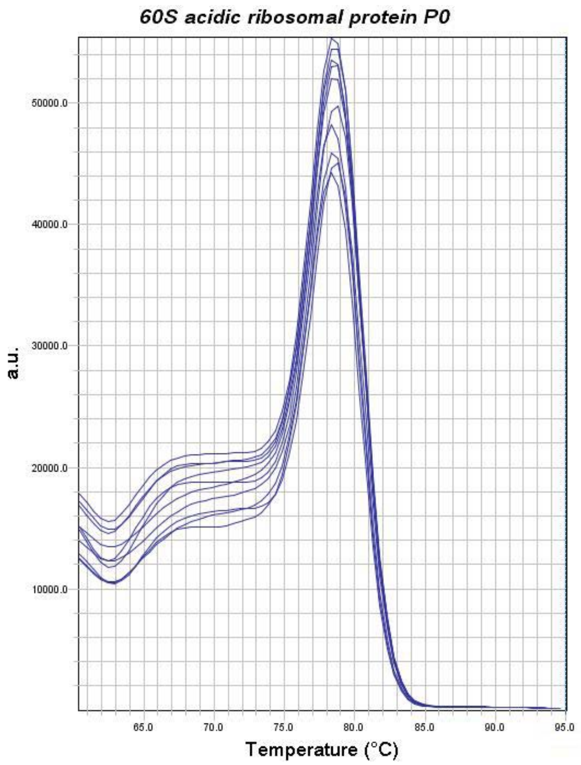
60S acidic ribosomal protein P0
50000.0
40000.0
30000.0
20000.0
10000.0
a.u.
65.0
70.0
75.0
80.0
85.0
90.0
95.0
Temperature (°C)

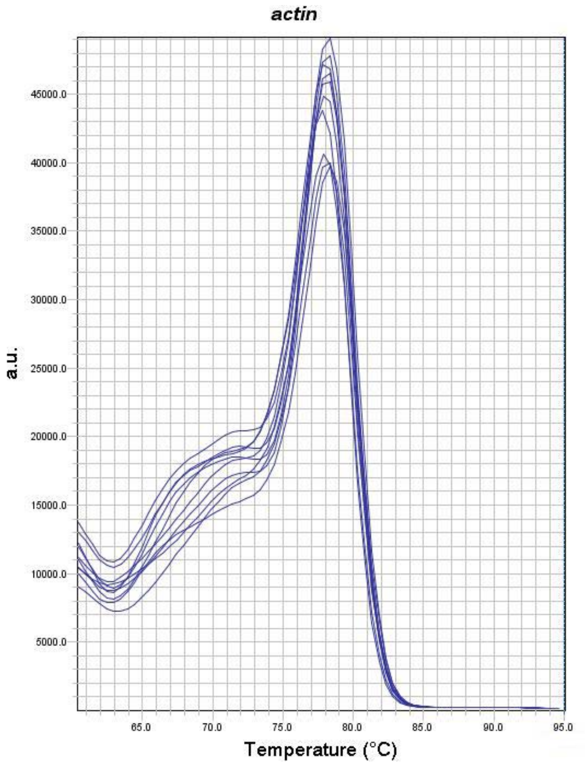
actin
45000.0
40000.0
35000.0
30000.0
25000.0
20000.0
15000.0
10000.0
5000.0
a.u.
65.0
70.0
75.0
80.0
85.0
90.0
95.0
Temperature (°C)

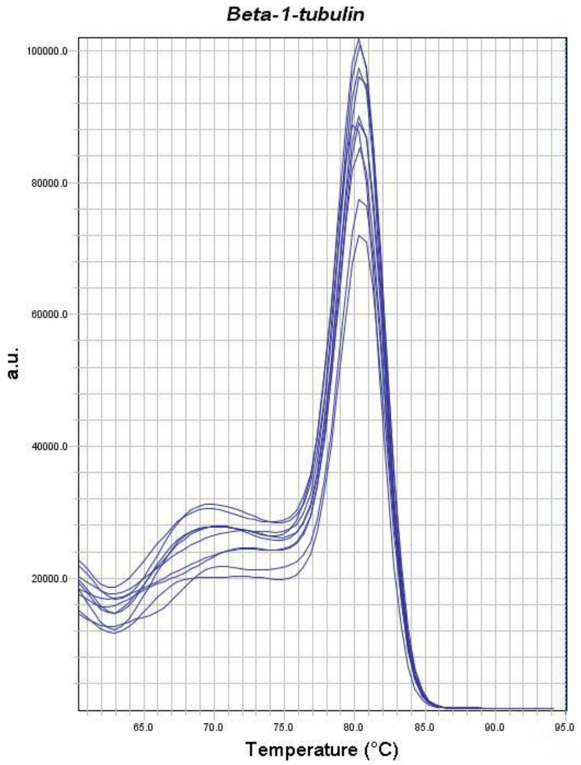
Beta-1-tubulin
100000.0
80000.0
60000.0
40000.0
20000.0
a.u.
65.0
70.0
75.0
80.0
85.0
90.0
95.0
Temperature (°C)

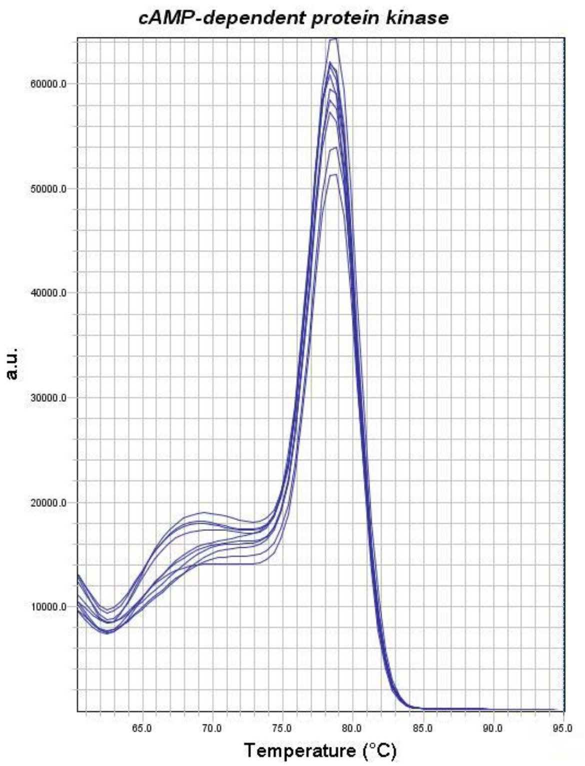
cAMP-dependent protein kinase
60000.0
50000.0
40000.0
30000.0
20000.0
10000.0
a.u.
65.0
70.0
75.0
80.0
85.0
90.0
95.0
Temperature (°C)

Glyceraldehyde-3-phosphate dehydrogenase 1

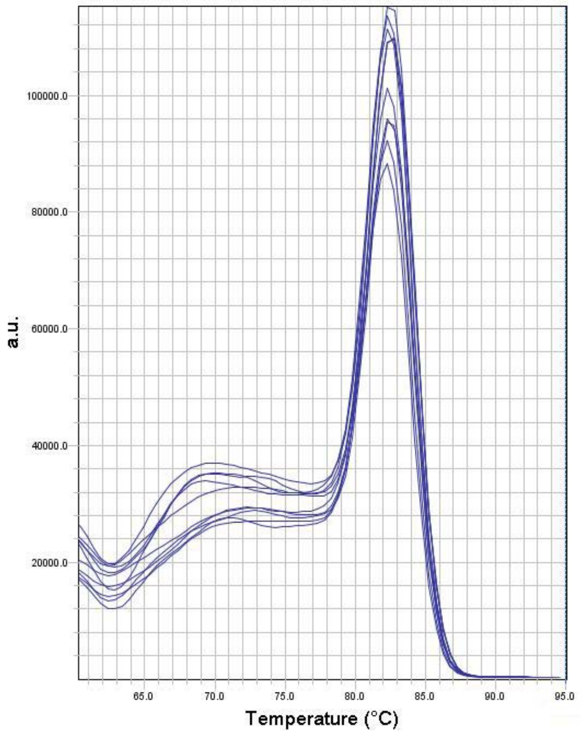

Glutathione S-transferases 1-1

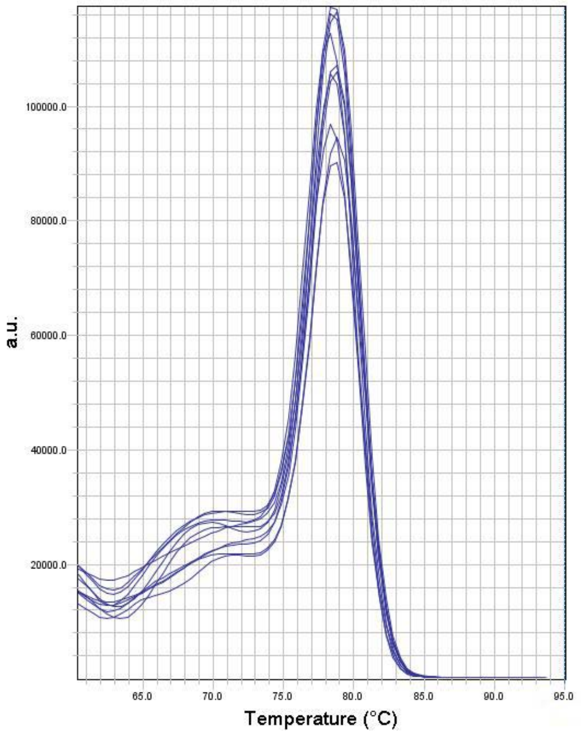

attacin-2

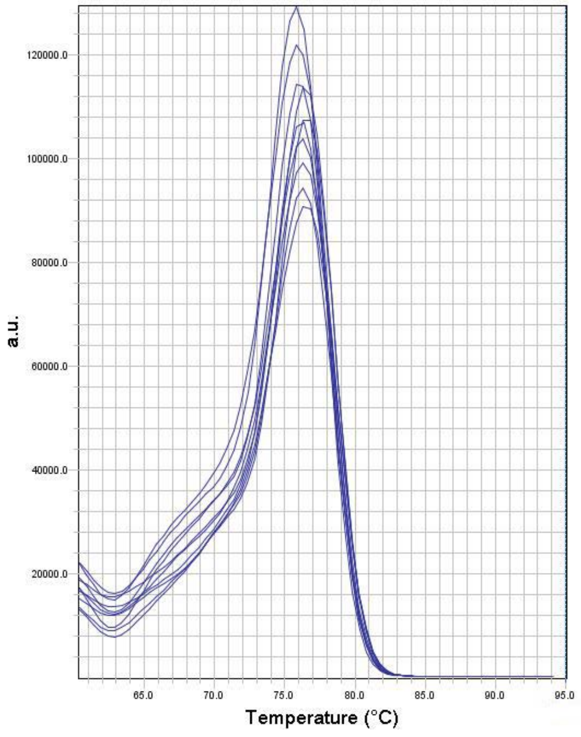

defensin-1

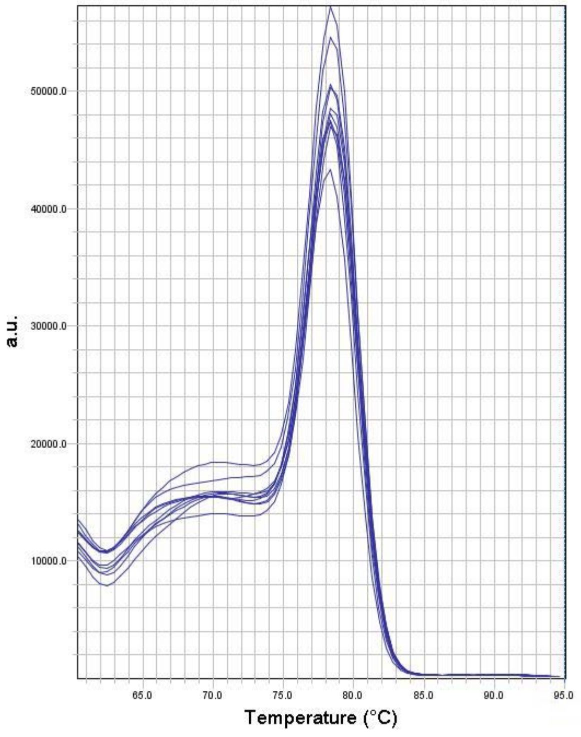

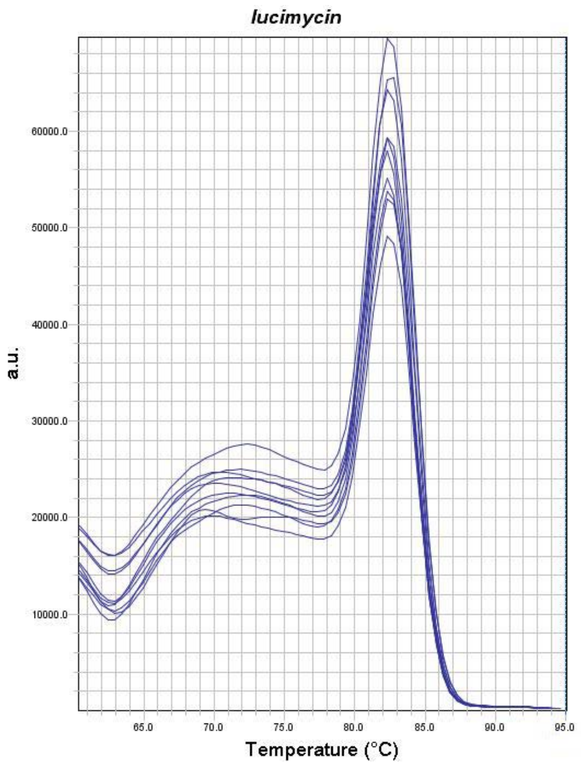
lucimycin
a.u.
60000.0
50000.0
40000.0
30000.0
20000.0
10000.0
65.0
70.0
75.0
80.0
85.0
90.0
95.0
Temperature (°C)
