# Supplementary material for: Selection and Evaluation of Tissue Specific Reference Genes in Lucilia sericata during an Immune Challenge
Source: PLoS One. 2015 Aug 7;10(8):e0135093. doi: 10.1371/journal.pone.0135093 (PMC4529112; doi:10.1371/journal.pone.0135093)
Supplement: S1 Table — (DOCX) [file pone.0135093.s003.docx]

**S1 Table.** **Normfinder intergroup variation for all candidate genes.**

| Gene | Larvae | Midgut | Hindgut | Salivary glands | Crop | Fat body | Nerve ganglion |
| --- | --- | --- | --- | --- | --- | --- | --- |
| *18S rRNA* | 0.001 | 0.000 | 0.001 | 0.003 | 0.001 | 0.000 | 0.000 |
| *28S rRNA* | 0.003 | 0.005 | 0.003 | 0.005 | 0.001 | 0.017 | 0.006 |
| *RPS3* | 0.000 | 0.001 | 0.000 | 0.000 | 0.000 | 0.000 | 0.001 |
| *EF1α* | 0.001 | 0.000 | 0.000 | 0.002 | 0.001 | 0.001 | 0.001 |
| *RPLP0* | 0.000 | 0.000 | 0.000 | 0.002 | 0.001 | 0.000 | 0.000 |
| *actin* | 0.000 | 0.000 | 0.000 | 0.001 | 0.000 | 0.003 | 0.000 |
| *β-tubulin* | 0.001 | 0.000 | 0.001 | 0.001 | 0.000 | 0.000 | 0.001 |
| *PKA* | 0.001 | 0.000 | 0.000 | 0.000 | 0.000 | 0.000 | 0.000 |
| *GAPDH* | 0.000 | 0.000 | 0.000 | 0.000 | 0.001 | 0.002 | 0.001 |
| *GST1* | 0.000 | 0.000 | 0.001 | 0.000 | 0.001 | 0.002 | 0.000 |
