# Supplementary material for: Selection and Evaluation of Tissue Specific Reference Genes in Lucilia sericata during an Immune Challenge
Source: PLoS One. 2015 Aug 7;10(8):e0135093. doi: 10.1371/journal.pone.0135093 (PMC4529112; doi:10.1371/journal.pone.0135093)
Supplement: S2 Table — (DOCX) [file pone.0135093.s004.docx]

**S2 Table. Normfinder intragroup variation for all canditate genes.**

| Gene | Larvae | Midgut | Hindgut | Salivary glands | Crop | Fat body | Nerve ganglion |
| --- | --- | --- | --- | --- | --- | --- | --- |
| *18S rRNA* | 0.032 | 0.018 | 0.066 | -0.053 | -0.016 | -0.100 | 0.053 |
| *28S rRNA* | -0.011 | -0.027 | 0.064 | -0.047 | 0.062 | -0.089 | 0.048 |
| *RPS3* | 0.028 | 0.015 | 0.008 | -0.023 | 0.012 | -0.012 | -0.028 |
| *EF1α* | -0.001 | -0.001 | 0.002 | -0.028 | 0.022 | -0.024 | 0.030 |
| *RPLP0* | -0.028 | -0.009 | -0.004 | -0.010 | 0.019 | -0.010 | 0.042 |
| *actin* | -0.103 | 0.016 | -0.006 | 0.084 | -0.099 | 0.094 | 0.012 |
| *β-tubulin* | 0.055 | 0.008 | -0.069 | -0.003 | -0.045 | 0.078 | -0.024 |
| *PKA* | -0.007 | 0.003 | -0.023 | -0.028 | 0.042 | 0.052 | -0.038 |
| *GAPDH* | -0.004 | -0.018 | 0.011 | 0.056 | 0.025 | 0.006 | -0.075 |
| *GST1* | 0.039 | -0.005 | -0.047 | 0.052 | -0.023 | 0.005 | -0.021 |
